# A Non-Covalent Dimer Formation of Quaternary Ammonium Cation with Unusual Charge Neutralization in Electrospray-Ionization Mass Spectrometry

**DOI:** 10.3390/molecules26195868

**Published:** 2021-09-28

**Authors:** Paulina Grocholska, Marta Kowalska, Robert Wieczorek, Remigiusz Bąchor

**Affiliations:** Faculty of Chemistry, University of Wrocław, F. Joliot-Curie 14, 50-383 Wrocław, Poland; paulina.grocholska@chem.uni.wroc.pl (P.G.); 292456@uwr.edu.pl (M.K.); robert.wieczorek@chem.uni.wroc.pl (R.W.)

**Keywords:** non-covalent dimers, gas phase, quaternary ammonium cation, charge neutralization, ESI-MS, hydrogen-deuterium exchange, computational analysis, DFT

## Abstract

Specific and nonspecific non-covalent molecular association of biomolecules is characteristic for electrospray-ionization mass spectrometry analysis of biomolecules. Understanding the interaction between two associated molecules is of significance not only from the biological point of view but also gas phase analysis by mass spectrometry. Here we reported a formation of non-covalent dimer of quaternary ammonium denatonium cation with +1 charge detected in the positive ion mode electrospray ionization mass spectrometry analysis of denatonium benzoate. Hydrogen deuterium exchange of amide and carbon-bonded hydrogens revealed that charge neutralization of one denatonium cation is the consequence of amide hydrogen dissociation. DFT (Density Functional Theory) calculations proved high thermodynamic stable of formed dimer stabilized by the short and strong N..H-N hydrogen bond. The signal intensity of the peak characterizing non-covalent dimer is low intensity and does not depend on the sample concentration. Additionally, dimer observation was found to be instrument-dependent. The current investigation is the first experimental and theoretical study on the quaternary ammonium ions dimer. Thus the present study has great significance for understanding the structures of the biomolecules as well as materials.

## 1. Introduction

Electrospray-ionization mass spectrometry (ESI-MS) has become a standard analytical technique for the investigation of molecules and complex mixtures. This technique has found a wide application in the determination of the elemental composition of analyzed molecules and allowed us to gain partial insight into the chemical structures of compounds under investigation due to the application of tandem mode (MS/MS) [1,2]. The combination of a high electric field, atmospheric pressure nebulizing gas and mild heating to effect desolvation, provides an approach for the analysis of non-covalent molecular association or conformational analysis. Since its introduction in 1988 by Fenn [3], it has been commonly used to investigate the gas-phase complexes of protein–protein, protein–DNA, protein–RNA, protein and small ligands, peptides and small molecules or of peptide-peptide [4,5]. Such studies are of great interest since biological activity often depends on their presence or absence. The most difficult aspect in the non-covalent complex formation in ESI-MS mode is to demonstrate unequivocally that the binding is specific. Nonspecific complexes are routinely observed in mass spectrometry which results from the ionization source parameters and analyte concentration. Although there is a lot of literature data concerning mentioned interaction in the gas phase during ESI-MS analysis [6,7,8], only a few reports have described the non-covalent complex formation between small organic molecules.

For example, the deprotonated and covalently bonded dimer of gingerols [9], stable proton-bridged dimer of ethanol [10] or (2-oxooxazolidin-4-ylmethyl)carbamic acid *tert*-butyl ester [11]. All of them are mostly dimer ions identified both in the positive and negative ion mode, denoted as [2M + H]^+^ or [2M − H]^−^.

Quaternary nitrogen atom containing compounds are a class of chemicals containing a permanent positive charge [12]. Its presence significantly increases the ionization efficiency of the analyzed compounds by ESI-MS, making their ultra-sensitive detection even at the attomolar level possible [13]. The introduction of the group containing quaternary nitrogen atom to the molecule of interest was also successfully applied as a derivatization strategy in ESI-MS analysis [14,15]. Additionally, in the MS/MS experiment, the presence of a fixed charge tag in the form of quaternary ammonium group is responsible for specific fragmentation patterns, which may facilitate the obtained mass spectra interpretation. The fragmentation mechanisms of compounds modified by quaternary ammonium groups were also studied and the obtained data revealed the competition between charge remote and charge directed mechanism [16]. Due to the specific activity of compounds containing quaternary nitrogen atom or atoms in the molecule, including tubocurarine, pancuronium, rocuronium, atracurium, decamethonium, suxamethonium or acetylcholine, the investigation of the possible dimer formation in the gas phase by these compounds is important for the explanation of their mechanism of action [17]. However, to the best of our knowledge, the non-covalent dimers of quaternary ammonium cations formed in the gas phase have not been described in the scientific literature. Therefore, there is a new field of scientific investigation which should be explored.

Recently, we developed the method of denatonium cation deuteration at the α-carbon atom based on the hydrogen-deuterium exchange (HDX) reaction in 1% solution of *N,N,N*-triethylamine in D_2_O at room temperature [18]. The obtained deuterated standard was also successfully applied in the quantitative LC-MS analysis of denatonium benzoate (Bitrex, *N,N*-diethyl-*N*-[(2,6-dimethylphenylcarbamoyl)methyl]benzylammonium benzoate), extremely bitter compound commonly used to denature industrial alcohols and to make potentially harmful household products unpalatable [19]. Denatonium cation is betaine derivative, which, based on our previous works, is able to undergo HDX at the α-carbon atom under basic conditions at room temperature [20,21,22]. The routine ESI-MS analysis of denatonium benzoate in the positive ionization mode revealed the presence of novel non-covalent dimer of denatonium cation with charge neutralization resulting in the formation of [2M − H]^+^ ion.

In the presented work, we described the gas phase formation of non-covalent dimer of quaternary ammonium denatonium cation which requires charge neutralization. The stability and geometry of the observed dimer was analyzed by the quantum mechanical calculations using density functional theory (DFT).

## 2. Results and Discussion

The aim of this work was to analyze and characterize the nature of the cationic dimer of denatonium cation with 1 +charge formed in the gas phase by using mass spectrometry and computational methods. The formation of such dimer requires charge neutralization of one denatonium cation which may be a consequence of the hydrogen atom dissociation from the analyzed molecule in the gas phase. Therefore, to determine the nature of the formed dimer and the deprotonation position the following ESI-MS and ESI-MS/MS analysis was performed.

### 2.1. Mass Spectrometry Analysis

**Figure 1:**
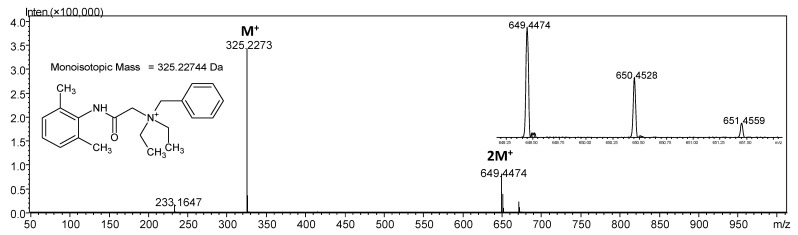
ESI-MS spectrum of denatonium cation in the positive ion mode. *m*/*z* range from 50 to 1000.

The mass spectrometrum of the denatonium benzoate in positive ion mode, as a compound containing quaternary nitrogen atom, is characterized by the signal at *m*/*z* 325.2273 which corresponds to the M^+^ denatonium ion (Figure 1).

Signal at *m*/*z* 233.1647 is the consequence of benzyl group dissociation in the gas phase. However, the analysis of the obtained mass spectrum revealed the presence of signal at *m*/*z* 649.4474. The analysis of the isotope pattern revealed that the ion characterized by this signal has two times more carbon atom than the native denatonium cation (Figure 1).

**Figure 2:**
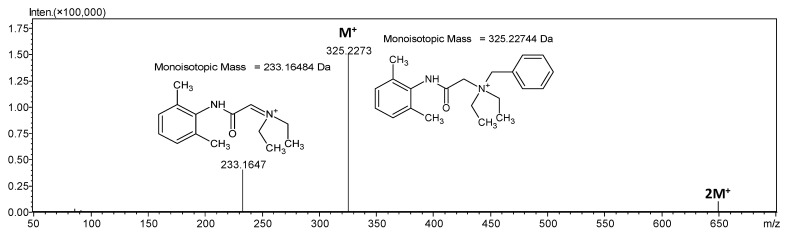
ESI-MS/MS spectrum of denatonium cation dimer in the positive ion mode. *m*/*z* range from 50 to 1000. Parent ion *m*/*z* 649.4475, collision energy 18–52 eV.

ESI-MS/MS analysis (Figure 2) of this ion revealed the formation of fragment ion at *m*/*z* 325.2273 which corresponds to the denatonium cation. Additionally, the signal at *m*/*z* 233.1647 corresponds to the dissociation of benzyl group from denatonium cation. It clearly confirms that the signal at *m*/*z* 649.4474 characterizes an ion consisting of two denatonium cations with the mass less by 1 Da than the mass of the theoretical dimer and 1 +charge.

**Figure 3:**
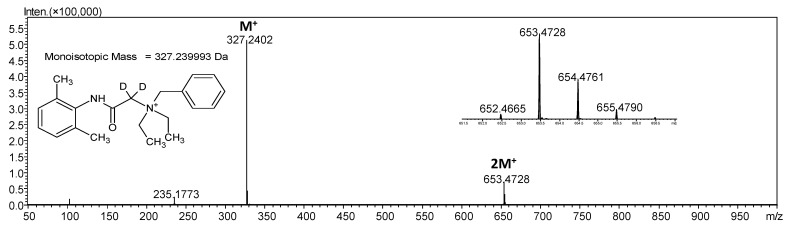
ESI-MS/MS spectrum of denatonium cation after hydrogen-deuterium exchange at the α carbon atom in the positive ion mode. *m*/*z* range from 50 to 1000.

It may suggest the neutralization of one denatonium cation within the dimer. This phenomenon may result from the proton dissociation from the one denatonium cation leading to the formation of a neutral molecule. To determine where the dissociation takes place, we decided to perform two other experiments using deuterated analogues of denatonium cation. In the first one, the isotopologue deuterated at the α carbon atom located between quaternary nitrogen and carbonyl carbon atoms was used to investigate the possibility of ylide formation which results in the neutralization of the denatonium cation. Such isotopologue was obtained according to the method previously described by us [18]. Briefly, the denatonium benzoate sample was dissolved in D_2_O and then *N,N,N*-triethylamine was added to obtain 1% solution with pD 12.4. After 30 min of incubation at room temperature the sample was lyophilized, redissolved in the mixture of H_2_O/MeCN and analyzed by ESI-MS. The obtained results are presented in Figure 3.

**Figure 4:**
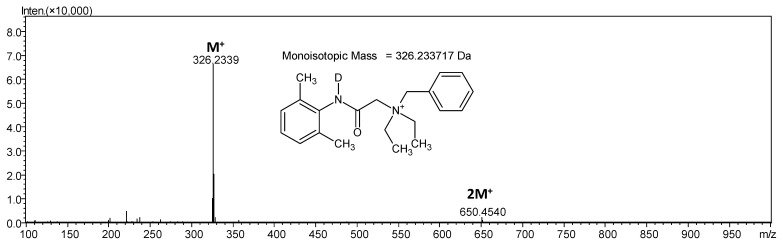
ESI-MS spectrum of denatonium cation after hydrogen-deuterium exchange of amide hydrogen in the positive ion mode. *m*/*z* range from 50 to 1000.

On the presented mass spectrum (Figure 3), obtained for the deuterated denatonium cation at the α carbon, the high intensity signal at *m*/*z* 327.2408 corresponds to the final product of the H/D exchange reaction. Additionally, the signal at *m*/*z* 653.4728 corresponding to the non-covalent dimer is present. If the formation of the discussed dimer in the gas phase occur due to the deprotonation at the α carbon atom within the denatonium cation the signal corresponding to such dimer should be characterized by the *m*/*z* at 652.4664 due to the loss of deuterium. The observed *m*/*z* value suggests that the neutralization results from the proton dissociation, not deuterium. There is a possibility of dissociation of benzyl hydrogen and amide hydrogen atoms. To find out which hydrogen is able to undergo dissociation we performed the ESI-MS analysis of the denatonium benzoate dissolved in D_2_O where only amide hydrogen atom was exchanged to deuterium. Amide hydrogen as a hydrogen connected with the heteroatom undergoes exchange with protons of the solvent within a few minutes whereas hydrogens bound to carbon atoms are not exchangeable under the applied conditions. The obtained results of the ESI-MS analysis are presented in Figure 4. Before the experiments with D_2_O all of the lines within the LC system were purged with D_2_O and the ion source was saturated with D_2_O for 30 min to remove the possible presence of H_2_O, which may cause the back exchange of the introduced deuteron at the amide nitrogen atom.

In the presented mass spectrum the high intens signal at *m*/*z* 326.2339 corresponds to the singly deuterated denatonium cation at the amide nitrogen. Additionally, the low intensity signal at *m*/*z* 650.4540, corresponding to the formed dimer, is present. Based on the obtained *m*/*z* ratio for the characteristic dimer formed in the gas phase it can be concluded that the dimer was formed due to the dissociation of the amide deuterium atom. The identified *m*/*z* value for such dimer (*m*/*z* 650.4540) is in good agreement with the calculated value (650.4538).

We also investigated the effect of used the mass spectrometer on the formation of denatonium cation dimer in the gas phase. In our study, we applied Apex-Qe 7T Bruker instrument (Bremen, Germany), Shimadzu LCMS-IT-TOF (Shimadzu, Kyoto, Japan) system equipped with Nexera X2 chromatographic module and LCMS-9030 qTOF Shimadzu (Shimadzu, Kyoto, Japan) device, equipped with a standard ESI source and the Nexera X2 system. The formation of non-covalent dimer of denatonium cation was observed only using the LCMS-9030 qTOF Shimadzu mass spectrometer. Additionally, we tested the possibility of dimer formation n using denatonium benzoate samples with different concentration (0.01 µmol/mL, 0.1 µmol/mL; 1 µmol/mL; 10 µmol/mL) and in all cases the intensity of signal corresponding to the formed dimer was at the same level. The performed analysis clearly confirmed that the formation of denatonium cation dimer in the gas phase with the charge neutralization of one molecule is possible and that the charge neutralization is related to the dissociation of amide hydrogen atom. The performed analysis is a step by step process, well planned, that gave the clear evidence of the observed phenomenon. It can be speculated that the location of the negative charge on the nitrogen atom may participate in the N..H-N hydrogen bond between two molecules of denatonium cation. To prove that we decided to perform computational analysis and determine the stability of the found dimer in the gas phase.

### 2.2. Computational Analysis

**Figure 5:**
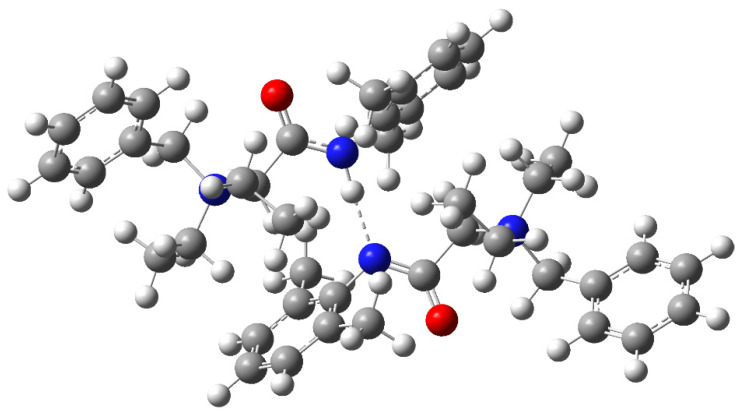
Calculated structure of non-covalent denatonium cation dimer with the strong N..H-N hydrogen bond.

The computational methods of theoretical chemistry have been used as a useful tool to predict structure and properties of organic and inorganic compounds [23,24,25,26,27] The Kohn-Sham molecular orbital studies on the analyzed non-covalent dimer as well as isolated subunits have been done on the DFT (Density Functional Theory) level of theory. The obtained results revealed that the observed dimer is thermodynamically stable (negative interaction energy, no imaginary frequencies), formed via a very strong N..H-N hydrogen bond (Figure 5).

The hydrogen bond is relatively short with N...N distance 2.850 Å and N..H length 1.812 Å. The N-H...N bond is very close being linear with N-H-N angle of 171.5 deg. All fully optimized structures of the complex and subunits can be found in Supplementary Materials.

Using the supermolecular approach, we have calculated the interaction energy of the complex (solvation energy included) ΔE = −51.6 kcal/mol the fact that the ion...molecule hydrogen bonds presented here belong to the most energetically efficient HB family and can reach up to ~−40 kcal/mol [28] the additional stabilization shall be achieved via stacking interactions, we estimate such contribution as high as ~20%.

The most frequently observed hydrogen bonds in organic compounds, as non-covalent interactions, include the O-H...O, N-H...O, C-H...O, O-H...N, and C-H...N. types [29,30]. The π -electron cloud of aromatic rings may also participate in interactions considered as similar to H-bonds, such as N-H...π, O-H...π, S-H...π, and C-H...π [31]. Hydrogen bonds of N-H...N type have been previously reported in small organic molecules structures [32,33] and proteins [30]. They are of great interest to chemists and biochemists, particularly due to their role in important biological systems stabilization and binding [34]. Recently, the competition between n→π(Ar)* and conventional hydrogen bonding (N-H...N) interactions in the complexes of 7-azaindole and fluorosubstituted pyridines has been reported [33]. In the presented investigation we identified only the strong N-H...N as non-covalent interaction stabilized the formed dimer.

## 3. Materials and Methods

### 3.1. Chemicals

All chemicals were used as supplied. Denatonium benzoate (≥98%), deuterium oxide (D_2_O, 99.9% purity), *N,N,N*-triethylamine (TEA), acetonitrile, formic acid (HCOOH) and water (LC/MS grade) were purchased from Sigma-Aldrich (Saint Louis, MO, USA).

### 3.2. Isotopic Exchange

Hydrogen-deuterium exchange was performed by dissolving 0.1 mg of denatonium benzoate in the 1% solution of TEA in D_2_O (200 μL) at room temperature (pD = 12.3). To observe only the introduced α-C deuterons the sample was lyophilized and redissolved in the mixture of water/MeCN (200 µL), incubated for 30 min and analyzed by ESI-MS.

### 3.3. Mass Spectrometry

All ESI-MS experiments were performed on the LCMS-9030 qTOF Shimadzu (Shimadzu, Kyoto, Japan) device, equipped with a standard ESI source and the Nexera X2 system. Analysis was performed in the positive ion mode between 50–1000 *m*/*z*. LCMS-9030 parameters: nebulizing gas-nitrogen, nebulizing gas flow—3.0 L/min, drying gas flow—10 L/min, heating gas flow—10 L/min, interface temperature 300 °C, desolvation line temperature—400 °C, detector voltage—2.02 kV, interface voltage—4.0 kV, collision gas – argon, collision energy was optimized between 10 and 30 eV. The injection volume was optimized depending on the intensity of the signals observed on the mass spectrum within the range of 0.1 to 1 μL. All obtained signals had a mass accuracy error in the range of 1 ppm. All of the used solvents were of LC-MS grade. In the experiments with native denatonium benzoate and for the α-C deuterated derivative samples were dissolved in water. The LC module was equipped with the water + 0.1% HCOOH as a mobile phase, eluent B: acetonitrile + 0.1% HCOOH. Flow rate—0.3 mL/min. Before the experiments with D_2_O all of the lines within LC system were purged with D_2_O and the ion source was saturated with D_2_O for 30 min to remove the possible presence of H_2_O. Only was used as an eluent in the LC module. The sample was dissolved in D_2_O. Flow rate—0.3 mL/min The obtained data were analyzed by LabSolutions software (Shimadzu, Kyoto, Japan).

### 3.4. Computational Analysis

The Kohn-Sham molecular orbital studies on non-covalent dimer as well as isolated subunits have been done on the DFT level of theory. Gaussian 16 C.016 [35] suite of programs using the ωB97X-D [36] long-range corrected hybrid density functional with damped atom-atom dispersion corrections was used with triple-ζ 6-311G(2d, 2p) basis set. All calculations: optimizations and energies have been done taking into account the solvent as a continuous medium PCM (polarizable continuum model) in IEFPCM (integral equation formalism for polarizable continuum model). The IEFPCM approximation describes a solvent as a homogeneous dielectric medium with electrical permeability (ε) equal to that of a pure solvent, and the cavity size is modeled for a solvent immersed molecule [37,38]. The presented structure was fully optimized with demanding convergence criteria (RMS Force = 1 × 10^−5^, RMS Displacement = 4 × 10^−5^, Max Force = 2 × 10^−5^, Max Displacement = 6 × 10^−5^) predefined as "opt = tight" in the Gaussian package, in atomic units. The graphic work has been done with GaussView 6.0.168 program [39].

## 4. Conclusions

In conclusion, we demonstrated the possibility of non-covalent dimer formation of quaternary ammonium denatonium cation in the gas phase during ESI-MS analysis. Dimer formation requires charge neutralization of one denatonium cation which is the consequence of the amide hydrogen deprotonation. The dimer is thermodynamically stable due to the presence of short, strong and practically linear N..H-N hydrogen bond. The detection of the observed non-covalent dimer ion appears to be instrument-dependent, as the protonated molecule of the compound could be observed in the mass spectrum from the qTOF mass spectrometer but was not detected in the spectra from IT-TOF or FT-ICR mass spectrometers. The abundance of the signal corresponding to the dimer was not concentration-dependent.

## Data Availability

Not applicable.

## Supplementary Materials

The following are available online. SM 1. The structure of the complex in Cartesian coordinates. SM 2. The structure of the subunit A in Cartesian coordinates. SM 3. The structure of the subunit B in Cartesian coordinates.

## Sample Availability

Samples of the analyzed compound are available from the authors.
