# Supplementary material for: A Non-Covalent Dimer Formation of Quaternary Ammonium Cation with Unusual Charge Neutralization in Electrospray-Ionization Mass Spectrometry
Source: Molecules. 2021 Sep 28;26(19):5868. doi: 10.3390/molecules26195868 (PMC8511985; doi:10.3390/molecules26195868)
Supplement: Supplementary file 1 [file molecules-26-05868-s001.zip › molecules-1358857-supplementary.pdf]

Article

A non-covalent dimer formation of quaternary ammonium cation with unusual charge neutralization in electrospray-ionization mass spectrometry

Paulina Grocholska , Marta Kowalska, Robert Wieczorek and Remigiusz Bąchor*

Faculty of Chemistry, University of Wrocław, F. Joliot-Curie 14, 50-383 Wrocław, Poland

* Correspondence: remigiusz.bachor@chem.uni.wroc.pl (R.B.); Tel.: +48 71 375 7218; Fax: +48 71 328 2348

SM 1. The structure of the complex in Cartesian coordinates.

C	-6.11176700	-0.86691100	-0.60998100
C	-7.15469900	0.05579500	-0.62965400
C	-8.17937200	-0.05688400	-1.55668900
C	-8.17713300	-1.10143300	-2.46911500
C	-7.15464400	-2.03887800	-2.44449900
C	-6.13107600	-1.92304400	-1.51819500
H	-7.18185100	0.86041900	0.09411500
H	-8.98547000	0.66362600	-1.55864600
H	-8.97880400	-1.19284200	-3.18878400
H	-7.15982200	-2.86613300	-3.14054000
H	-5.34222700	-2.66415600	-1.48780300
C	-4.99798700	-0.78124300	0.39238500
H	-5.35497400	-0.42935500	1.35606400
N	-3.84931300	0.15559400	0.01071000
C	-3.37424100	-0.18638300	-1.38430800
H	-3.18324300	-1.25591100	-1.37040100
H	-4.21568200	0.00545100	-2.04450300
C	-2.14226500	0.56763400	-1.84768900
H	-2.31004600	1.63643500	-1.96170300
H	-1.29516100	0.41119900	-1.18296500
H	-1.87157400	0.17248800	-2.82624800
C	-4.27960800	1.60254700	0.03280000
H	-5.03300700	1.70617100	-0.74560700
H	-3.40731600	2.18548400	-0.25300000
C	-4.80019200	2.11576500	1.36090400
H	-5.72080200	1.63205900	1.67954700
H	-4.05981000	2.02987200	2.15287500
H	-5.01217400	3.17676700	1.23685700
C	-2.73480700	-0.03974300	1.00757600
H	-3.19253800	-0.04605400	1.99462300
H	-2.09202100	0.82806700	0.93156600
C	-1.93692800	-1.34456400	0.83820400
O	-2.56234800	-2.39962200	0.66078400
N	-0.63533800	-1.15808000	0.96571300
C	0.18510400	-2.30635500	0.95315500
C	0.91219900	-2.60223300	2.11920700
C	0.35543700	-3.09820400	-0.19483400
C	1.79225100	-3.67708100	2.12449800
C	1.26229200	-4.15388100	-0.15783600
C	1.97774100	-4.45109700	0.99026800
H	2.32883700	-3.91667500	3.03445500
H	1.38911300	-4.76638400	-1.04231300
H	2.65942500	-5.29081400	1.00635100
C	-0.44006100	-2.84622600	-1.44365900

H	-1.44919800	-3.23903100	-1.31829200
H	-0.53571800	-1.77935800	-1.64689000
H	0.02112700	-3.32439200	-2.30711900
C	0.70916300	-1.77287800	3.35752100
H	0.93029000	-0.71742800	3.18024300
H	-0.32936900	-1.81532100	3.68737500
H	1.34073300	-2.12454100	4.17221100
H	-4.51140700	-1.74333500	0.53611800
C	6.14294300	0.69305200	-0.65475800
C	7.13458800	-0.28192300	-0.57941600
C	8.17221800	-0.30500600	-1.49807900
C	8.23534300	0.65427300	-2.49767100
C	7.26500200	1.64319100	-2.56934900
C	6.22798600	1.66324400	-1.65096200
H	7.11218000	-1.02180500	0.20984200
H	8.93750600	-1.06513500	-1.42601500
H	9.04745800	0.63959100	-3.21115100
H	7.32149700	2.40478000	-3.33450600
H	5.48165900	2.44641800	-1.69851000
C	5.01930800	0.75303600	0.33931100
H	5.34976300	0.46233700	1.33232300
H	4.59856500	1.75230300	0.39954900
N	3.82105600	-0.14864100	0.02378500
C	3.35675300	0.12662100	-1.39108800
H	3.21708000	1.20303000	-1.44986800
H	4.19149900	-0.13946800	-2.03328300
C	2.09896500	-0.60577500	-1.81385300
H	2.23551400	-1.68221200	-1.87864800
H	1.25358000	-0.40983900	-1.15726500
H	1.83543900	-0.24692300	-2.80815700
C	4.18196200	-1.61544400	0.14133000
H	4.91626200	-1.80351500	-0.63897500
H	3.27793800	-2.17384500	-0.09050500
C	4.70084000	-2.05510000	1.49542500
H	5.64426800	-1.58992400	1.77201600
H	3.97296200	-1.89464000	2.28719800
H	4.86525700	-3.12980300	1.43885000
C	2.72723400	0.14879200	1.01015300
H	3.18228500	0.19266700	1.99715900
H	2.03866900	-0.69025500	0.99189800
C	2.00454600	1.47772900	0.77525400
O	2.61689000	2.51224400	0.56798600
H	0.22543800	0.43668600	0.99824500
N	0.67160000	1.37296100	0.87105900
C	-0.19830600	2.50095800	0.82674800
C	-0.92098400	2.80417300	1.98529000
C	-0.35917200	3.22980300	-0.35330600
C	-1.82504100	3.85761600	1.94334300
C	-1.27897400	4.27490900	-0.35542600
C	-2.00668900	4.58838300	0.77984700
H	-2.38256700	4.11321800	2.83509300
H	-1.41550400	4.85375500	-1.25975600
H	-2.70810600	5.41156600	0.76176600
C	0.43746700	2.91999500	-1.58809100
H	1.44929000	3.31242900	-1.48398100
H	0.51744500	1.84520000	-1.75203400
H	-0.02017400	3.36774000	-2.46826500
C	-0.71561500	2.00426100	3.24223400
H	-1.36858600	2.35710000	4.03796200
H	-0.91020900	0.94195100	3.08075100
H	0.31523000	2.08750800	3.58967500

SM 2. The structure of the subunit A in Cartesian coordinates.

C	-3.34976000	-0.80397400	0.13831900
C	-4.55139100	-0.39432100	0.71208400
C	-5.76263500	-0.76890800	0.15273900
C	-5.78377400	-1.56657100	-0.98190500
C	-4.59321500	-2.00005400	-1.54726700
C	-3.38319500	-1.62459000	-0.98695300
H	-4.54704900	0.20508000	1.61405700
H	-6.68879300	-0.44922100	0.60925900
H	-6.72821100	-1.86362100	-1.41595600
H	-4.60721500	-2.64057800	-2.41781100
H	-2.45753100	-1.98502700	-1.41768100
C	-2.03206900	-0.42519900	0.74657200
H	-2.10809100	-0.33302200	1.82676400
H	-1.26379800	-1.15417100	0.51119300
N	-1.45267300	0.91398100	0.25829600
C	-1.35966100	0.89689800	-1.26045600
H	-0.93572900	-0.06601700	-1.52222600
H	-2.38653900	0.93317300	-1.61312500
C	-0.53221200	2.01227700	-1.86925300
H	-0.90578900	3.00891800	-1.64188000
H	0.51878100	1.95359400	-1.59115800
H	-0.58059100	1.89292800	-2.95043500
C	-2.35424900	2.06379300	0.65735100
H	-3.32239500	1.83804000	0.21812200
H	-1.95868900	2.94596800	0.16146300
C	-2.48577200	2.32436000	2.14544500
H	-2.85641900	1.46444000	2.69931600
H	-1.55647600	2.66134900	2.60032900
H	-3.21299200	3.12518700	2.27097700
C	-0.10987700	1.10675600	0.89195500
H	-0.21841100	0.90576300	1.95594000
H	0.16156100	2.15389500	0.77356800
C	0.99937700	0.19463600	0.34666500
O	0.76251200	-0.86843200	-0.18826900
H	2.32525500	1.61040400	0.94518000
N	2.23350100	0.67924500	0.57447100
C	3.44029600	-0.01331100	0.22413600
C	3.79582000	-1.16854800	0.91856600
C	4.22920100	0.52477800	-0.79288400
C	4.98300700	-1.79573300	0.55817100
C	5.41211800	-0.12682100	-1.11364800
C	5.78475500	-1.28127900	-0.44548800
H	5.28274600	-2.69463500	1.08009100
H	6.04166500	0.27163500	-1.89795400
H	6.70718600	-1.78083300	-0.70707000
C	3.80533400	1.76662000	-1.52860200
H	2.84271900	1.62028700	-2.02205800
H	3.70942900	2.62654900	-0.86156300
H	4.53341800	2.02901500	-2.29256900
C	2.93400200	-1.72977300	2.01368500
H	2.59488200	-0.94799900	2.69504900
H	2.05152000	-2.21623500	1.59575400
H	3.48306800	-2.46622300	2.59581500

SM 3. The structure of the subunit B in Cartesian coordinates.

C	3.27627900	-0.68313400	-0.45728200
C	4.54059600	-0.12089300	-0.61404500
C	5.67130600	-0.78239000	-0.15954800
C	5.54968300	-2.02194800	0.45031400
C	4.29703400	-2.60163200	0.59296800
C	3.16899500	-1.93787400	0.13837500
H	4.64741600	0.83616400	-1.10983900
H	6.64704200	-0.33470700	-0.29034600
H	6.43069300	-2.54108500	0.80232600
H	4.19880200	-3.57586200	1.05183300
H	2.19219700	-2.39460200	0.23502800
C	2.04045700	0.00493600	-0.95937500
H	2.24228200	0.55558400	-1.87407500
H	1.22539900	-0.69650100	-1.14504500
N	1.41682800	1.01187200	0.00293900
C	1.20526600	0.35071000	1.35051800
H	0.68582800	-0.57725800	1.12837800
H	2.19741000	0.12323100	1.73344000
C	0.40726700	1.16865600	2.34972200
H	0.92116800	2.07229900	2.67480700
H	-0.58139200	1.41993000	1.97046800
H	0.26659800	0.54557200	3.23232000
C	2.29816200	2.21300500	0.19431700
H	3.23006200	1.84443200	0.61960200
H	1.80958200	2.83016900	0.94262600
C	2.56415600	3.04546800	-1.04569200
H	3.08106200	2.49218500	-1.82724700
H	1.65091500	3.46511800	-1.46162000
H	3.20642900	3.87612600	-0.75537000
C	0.08436300	1.42728900	-0.58582500
H	0.26183000	1.63341500	-1.63984500
H	-0.23329200	2.33887000	-0.09050400
C	-1.01736300	0.35251800	-0.45371000
O	-0.70339900	-0.83865300	-0.69377200
N	-2.14610700	0.89688900	-0.10834400
C	-3.30189500	0.10174700	-0.08257000
C	-3.86293400	-0.41771800	-1.26061000
C	-3.96025900	-0.08484200	1.14187700
C	-5.06097900	-1.11721900	-1.18816100
C	-5.15471200	-0.79203600	1.17775200
C	-5.70980300	-1.31173300	0.02012000
H	-5.49164000	-1.51470900	-2.09998000
H	-5.65479700	-0.93559700	2.12865300
H	-6.64305600	-1.85813700	0.05793500
C	-3.35699600	0.47183400	2.40118500
H	-2.42606100	-0.04335000	2.64983900
H	-3.11222800	1.52827900	2.27832700
H	-4.03880600	0.36294000	3.24470000
C	-3.17801400	-0.20737800	-2.58093600
H	-2.91251100	0.84394900	-2.71234400
H	-2.25194700	-0.78346600	-2.62463400
H	-3.82049000	-0.50928500	-3.40777600
